# Modulation of Cancer Traits by Tumor Suppressor microRNAs

**DOI:** 10.3390/ijms14011822

**Published:** 2013-01-16

**Authors:** Ioannis Grammatikakis, Myriam Gorospe, Kotb Abdelmohsen

**Affiliations:** Laboratory of Genetics, National Institute on Aging-Intramural Research Program, National Institutes of Health, 251 Bayview Blvd, Baltimore, MD 21224, USA; E-Mails: yannis.grammatikakis@nih.gov (I.G.); gorospem@mail.nih.gov (M.G.)

**Keywords:** post-transcriptional gene regulation, oncomiR, tumor suppressor microRNA, senescence, carcinogenesis

## Abstract

MicroRNAs (miRNAs) are potent post-transcriptional regulators of gene expression. In mammalian cells, miRNAs typically suppress mRNA stability and/or translation through partial complementarity with target mRNAs. Each miRNA can regulate a wide range of mRNAs, and a single mRNA can be regulated by multiple miRNAs. Through these complex regulatory interactions, miRNAs participate in many cellular processes, including carcinogenesis. By altering gene expression patterns, cancer cells can develop specific phenotypes that allow them to proliferate, survive, secure oxygen and nutrients, evade immune recognition, invade other tissues and metastasize. At the same time, cancer cells acquire miRNA signature patterns distinct from those of normal cells; the differentially expressed miRNAs contribute to enabling the cancer traits. Over the past decade, several miRNAs have been identified, which functioned as oncogenic miRNAs (oncomiRs) or tumor-suppressive miRNAs (TS-miRNAs). In this review, we focus specifically on TS-miRNAs and their effects on well-established cancer traits. We also discuss the rising interest in TS-miRNAs in cancer therapy.

## 1. Introduction

MicroRNAs (miRNAs) are small non-coding RNAs that regulate gene expression post-transcriptionally. In mammalian cells, miRNAs suppress the expression of proteins encoded by target mRNAs with which miRNAs interact with incomplete complementarity; typically occurring with the 3′-untranslated region (UTR) of the target mRNA and leading to its degradation and/or its translational suppression [1,2]. The target mRNAs of a given miRNA can be predicted with a relatively high degree of success using sequence algorithms that identify homologies between the “seed” region of the miRNA and the complementary site on the target mRNA. These interactions typically occur between the miRNA seed region and a complementary sequence in the 3′-untranslated region (UTR) of the target mRNA, but they may also take place with the mRNA coding region or 5′UTR, as well as with seedless miRNAs [3,4]. Through their influence on subsets of target mRNAs, miRNAs have emerged as critical regulators of numerous cellular processes, including cell division, differentiation, senescence and apoptosis [5–7]. Thus, dysregulated miRNA expression can have a profound impact upon the cell fate and can lead to the development of pathologies like cancer [8]. Cancer cells typically display special miRNA signatures distinct from those of normal cells; accordingly, some miRNAs are currently used as cancer biomarkers [9–13]. There is strong interest in studying the patterns of miRNAs in cancer, since they can directly influence the production of tumor suppressor proteins and oncoproteins and hence affect tumor development and response to therapy. Some miRNAs are selectively increased in cancer cells, but more often, miRNAs show decreased expression in cancer cells [14–16]. Considering their influence on the cancer cell phenotype, some miRNAs are considered to be oncogenic (oncomiRs), and other miRNAs are considered to be tumor-suppressive (TS-miRNAs).

As proposed by Hanahan and Weinberg, normal cells acquire a number of characteristics as they transform into cancerous cells [17]. For cancer cells to thrive, they must remain in a proliferative state, survive despite adverse surrounding conditions, elicit local angiogenesis, invade other tissues, metastasize and evade recognition by the body’s immune system. Numerous TS-miRNAs are downregulated in cancer tissues; upon re-expression, they suppress various processes relevant to tumorigenesis, including proliferation, apoptosis and migration. In this review, we focus on the influence of TS-miRNAs on the hallmark traits acquired by cancer cells. We consider TS-miRNAs broadly as miRNAs targeting mRNAs that encode proteins, which enable cancer traits. We also discuss the growing efforts to exploit TS-miRNAs in cancer therapy.

## 2. TS-miRNAs that Suppress Cell Growth and Proliferation

For cells to become a tumor, they must proliferate in order to augment the size of the transformed cell population. Sustained cancer cell proliferation normally requires a continuous supply of proliferative signals. For example, the levels and/or function of proteins that promote cell cycle progression, such as cyclins and cyclin-dependent kinases (cdks), are commonly elevated in cancer cells and result in shorter division periods [18]. In addition, there is increased expression of several other proteins that promote cancer cell growth and proliferation, including those required to sustain proliferative signals, like B cell CLL/lymphoma 2 (Bcl-2), B-cell lymphoma-extra large (Bcl-xL), sirtuin 1 (SirT1), high-motility group AT-hook gene 1 (HMGA1) and other factors discussed below. Factors that stimulate cancer cell growth include the epidermal growth factor (EGF), vascular endothelial growth factor (VEGF) and platelet-derived growth factor (PDGF) [19]. In this section, we review examples of TS-miRNAs that block or reduce cancer cell proliferation and growth.

Several TS-miRNAs suppress the expression of one or more of these proliferation enhancers and thus block the growth of different cancers. For example, miR-34a represses the production of Bcl-2 and SirT1, two proteins displaying high levels of expression in cancer cells and implicated in promoting proliferation and cell survival [20–22], although miR-34a does not affect Bcl-2 expression levels in all cell systems [23]. In agreement with the finding that transgenic mice overexpressing AIB1 (Amplified In Breast Cancer 1) developed mammary epithelial cell proliferation, hyperplasia and tumorigenesis, low levels of miR-17-5p in breast cancer correlated with increased expression of the mRNA encoding AIB1, and conversely, miR-17-5p overexpression suppressed cell proliferation [24–26]. Also in breast cancer cells, miR-125a was shown to target the RNA-binding protein HuR, which is essential for proliferation and broadly enhances cancer traits [27,28]. Another miRNA targeting HuR, miR-519 suppresses cell proliferation and inhibits tumorigenesis [29,30]. The effect of miR-519 on cell proliferation was extended to ovarian, lung and kidney tumors, where the low abundance of miR-519 correlated inversely with HuR protein levels [30]. In breast cancer, the promoter of miR-125b was methylated and silenced, which allowed the levels of the miR-125b target oncoprotein (Ets1) to rise. In agreement with the finding that restoring miR-125b expression inhibited breast cancer cell proliferation by blocking Ets1, high levels of Ets1 correlated with poor patient prognosis [31]. miR-125b was also able to block cell proliferation in hepatocellular carcinoma (HCC) by targeting the anti-apoptotic protein Bcl-2 [32]. The influence of miR-125b on tumorigenesis may be cell- and tissue-specific, since it promoted malignant transformation of different hematopoietic lineages in mice and was upregulated in acute megakaryoblastic leukemia [33,34]. By contrast, the levels of miR-1 were drastically low in thyroid adenomas and carcinomas, and in these cells, miR-1 levels were inversely correlated with those of cyclin D1, required for G1/S transition [35].

miR-1 is epigenetically silenced in primary and distant metastatic human prostate tumors. Overexpression of miR-1 disrupted cell cycle progression, which subsequently led to growth inhibition and suppression of prostate cancer xenograft growth [36]. miR-1 expression levels are also reduced in primary human HCC compared with normal liver tissues, and ectopic expression of miR-1 in HCC cells inhibited cell growth and reduced replication potential and clonogenic survival. These effects were associated with inhibition of cell cycle progression and induction of apoptosis [37]. In addition, miR-1 is downregulated in human primary lung cancer tissues and cell lines, and its overexpression in lung carcinoma cell lines A549 and H1299 reduced cell growth and tumor formation in nude mice. These effects were associated with reduced expression of oncogenes, including the receptor tyrosine kinase MET and serine/threonine-protein kinase Pim-1, which are often upregulated in lung cancer and are involved in cell growth and proliferation [38].

miR-28 expression was lower in colorectal cancer than in normal colon, and its restoration led to an inhibition of cell proliferation [39]. Expression of miR-205 was silenced in prostate cancer; since miR-205 transcriptionally activated the expression of tumor suppressors interleukin (IL)-24 and IL-32, miR-205 reintroduction led to the re-expression of IL-24 and IL-32, triggering apoptosis and growth arrest [40]. miR-296 downregulated HMGA1 expression in prostate cancer cells, in turn reducing cell proliferation [41]. In gastric cancer, miR-148b inhibited cell proliferation *in vitro* and *in vivo* by lowering the levels of cholecystokinin-B receptor (CCKBR), a protein that promotes tumorigenesis [42].

miR-135a suppressed gastric cell proliferation at least partly by reducing the production of the cytoplasmic tyrosine kinase JAK2 (Janus kinase 2), which influences cell proliferation through its downstream signaling effectors STAT3, cyclin D1 and Bcl-XL [43]. miR-146a suppressed cell proliferation in extranodal NK/T cell lymphoma (NKTL) and was proposed to function as a general TS-miRNA by targeting genes involved in cell proliferation (reviewed in [44,45]). In colon and breast cancer cells, miR-145 suppressed tumor growth indirectly by targeting p70S6K1 (required for expression of VEGF and hypoxia-inducible factor 1 (HIF-1)) and directly by targeting VEGF-A [46,47]. In glioma cells, miR-128 suppressed tumor growth by targeting EGF and PDGF receptors, thus inhibiting mitogenic growth signals [48].

Other proposed TS-miRNAs include miR-101, miR-143, miR-24, miR-133a, miR-133b, miR-138, miR-216b, miR-155, miR-138, miR-508-3p and miR-509-3p, since they suppressed cell proliferation and growth in different cancers, such as bladder transitional cell carcinoma (TCC), laryngeal squamous cell carcinoma (LSCC), esophageal squamous cell carcinoma (ESCC) and melanoma [49–54]. In sum, altered miRNA levels in cancer cells can help to promote cancer progression by increasing cell proliferation. In some instances, the restoration or overexpression of these miRNAs was effective in suppressing cancer proliferation both *in vitro* and *in vivo*.

## 3. TS-miRNAs that Enhance Cell Death

The survival of cancer cells can be enhanced through the accumulation of genetic mutations that lower the levels of some tumor suppressors, like p53 (which triggers growth arrest, senescence and apoptosis), or by altering the expression of tumor suppressor-regulatory proteins, like Mdm2, which enhances p53 degradation [55–58]. In addition, cancer cell survival can be improved by the expression of anti-apoptotic proteins, such as members of the Bcl-2 family. Thus, TS-miRNAs can enhance cancer cell death by regulating anti-apoptotic factors. For example, p53 is induced in response to DNA-damaging agents, such as ionizing radiation, in turn transcriptionally enhancing miR-34a expression. Indeed, irradiated chronic lymphocytic leukemia (CLL) showed higher levels of miR-34a, leading to the induction of Bax and p21, but not Puma. These findings suggest that functional p53 increases miR-34a expression upon DNA damage and that, in turn, miR-34a may block cancer cell growth by triggering cell cycle arrest or apoptosis through the suppression of target proteins, like SirT1, Bcl-2 or cyclin D1 [59]. Since miR-34a targets include the anti-apoptotic protein Bcl-2, baculoviral IAP repeat-containing 3 (BIRC3) and decoy receptor 3 (DcR3), the combined effects of upregulating p53 and downregulating miR-34a targets strongly enhance cancer cell death [20,60–62]. While Bcl-2 inhibits the formation of the apoptosome by blocking the release of cytochrome c from mitochondria, BIRC3 inhibits apoptosis by interacting with the tumor necrosis factor (TNF) receptor-associated factors TRAF1 and TRAF2 [63]. DcR3 is highly expressed in many tumors and suppresses apoptosis by acting as a decoy receptor for ligands that would otherwise trigger cell death by binding to proteins, such as Fas receptor [64]. In cultured brain tumor (glioma) stem cells, miR-34a induced apoptosis through the inhibition of the oncogenic, pro-survival factors c-Met, Notch-1 and Notch-2, which are important for cell survival [65]. miR-34a also targets mRNAs encoding cell cycle regulators necessary for cell division, such as Cyclin D1, Cyclin E2, Cdk4, Cdk6 and E2F [66,67], indicating that cell cycle arrest is also a powerful mechanism through which miR-34a suppresses tumorigenesis. In HCC, miR-125b inhibits cell proliferation, as explained above, and promotes apoptosis by lowering Bcl-2 expression [32], while miR-1 promotes apoptosis, as mentioned above [37]. In glioma cell lines, miR-181d suppressed tumor growth by lowering the levels of both K-Ras and Bcl-2, while in glioblastoma cells, miR-451 reduced the levels of Cyclin D1 and Bcl-2, causing growth arrest and enhancing cell death [68,69]. In cervical cancer cells, miR-519 increased the levels of p53 and p21, causing cell cycle arrest and cellular senescence, and it induced DNA damage by lowering the abundance of several DNA repair enzymes [70,71]. In conclusion, reducing TS-miRNA activity in cancer cells can enable cancer progression by inducing cell survival and reducing apoptosis. Conversely, overexpression of these TS-miRNAs can restore the sensitivity of cancer cells to death signals; in some cases, it can enhance cellular senescence.

## 4. TS-miRNAs that Suppress Angiogenesis

Cancer cells need to secure a constant supply of nutrients in order to thrive and expand. Growth of the tumor requires enhanced angiogenesis, a process that generates new blood vessels to deliver nutrients and oxygen. This cancer trait is elicited by major angiogenesis factors: vascular endothelial growth factor (VEGF), fibroblast growth factor 2 (FGF-2) and platelet-derived growth factors (PDGF)-B and C [72,73]. The transcription factor HIF-1α is highly expressed in hypoxic conditions and transcriptionally increases VEGF expression in adaptive and neoplastic angiogenesis [74]. In addition, matrix metalloproteinases (MMPs) facilitate angiogenesis by assisting in the degradation of the extracellular matrix (ECM), a process that releases pro-angiogenic growth factors like VEGF and FGF-2 [75,76].

Several TS-microRNAs inhibit this tumor trait. miR-145 suppresses tumor angiogenesis, growth and invasion by lowering the production of the oncogene VEGF-A and by suppressing the expression of the serine/threonine kinase p70S6K1, which promotes HIF-1α and VEGF activities in colon cancer cells [46,47]. Similarly, suppression of p70S6K1 by miR-128 in glioma cell lines led to the downregulation of HIF-1α and VEGF and to the subsequent inhibition of angiogenesis *in vivo* [77]. In glioma cell lines and tumors, miR-205 was further identified as a direct repressor of VEGF-A and, hence, a suppressor of angiogenesis [78]. miR-519c reduced HIF-1α levels, suppressing tumor formation and angiogenesis in lung and breast cancer cell lines, while it also inhibited the biosynthesis of HuR, which regulates numerous cancer traits, including angiogenesis [27,29,30,70,71,79,80]. In breast cancer cells, miR-340 suppressed tumor cell migration and invasion by lowering the abundance of c-Met, a factor that promotes expression of MMP-2 and MMP-9; MMP-2 was additionally repressed by miR-29b in HCC cells, attenuating HCC cell invasiveness and angiogenic activity [81,82]. Finally, miR-9 lowered MMP-14 in neuroblastoma cells, causing an attenuation of tumor growth, metastasis and angiogenesis *in vivo* [83]. In sum, downregulation of several TS-miRNAs in cancer cells can augment angiogenesis by promoting the expression of pro-angiogenic factors and enzymes that degrade the ECM. Together, they facilitate the generation of new blood vessels that support the cancer tissue with nutrients and oxygen. Restoration of some of these miRNAs has been shown to suppress the expression of these factors and to attenuate angiogenesis.

## 5. TS-miRNAs that Enhance Immune Recognition

The immune system is a vital barrier against tumor formation and progression. Constant surveillance by the immune cells leads to eradication of virus-induced and some forms of non-virus-induced tumors. Immunodeficient mice, particularly mice lacking natural killer cells, CD4^+^ Th1 helper T-cells or CD8^+^ cytotoxic T-lymphocytes, developed tumors more frequently and faster than control animals [84,85]. A number of tumor-suppressive mechanisms have been identified that enable cancer cells to escape immune system surveillance and render them refractory to immune attack. Tumors can evade immune recognition by overproducing inhibitors of T-cell responses, such as galectins (small proteins involved in immune response, inflammation and tumorigenesis [86]) and indoleamine 2,3-dioxygenase (IDO) [87,88], as well as by increasing the production of immune suppressive cytokines, like transforming growth factor beta (TGF-β) and interleukin 10 (IL-10) [89,90]. Tumors can also suppress proinflammatory signals by activating the signal transducer and activator of the transcription 3 (STAT3) pathway, thereby blocking tumor-specific T-cell responses [91]. Other immune-suppressive mechanisms involve the downregulation of natural killer cell receptor protein G2D (NKG2D) to reduce lymphocyte-mediated cytotoxicity and the generation of active immune-suppressive cells, such as myeloid-derived suppressor cells (MDSCs) [92,93].

TS-miRNAs can inhibit this tumor trait by facilitating the immune response, diminishing immune-suppressive mechanisms and/or suppress the STAT3 pathway. In this regard, miR-322 suppressed the expression of galectin-3 [94], while miR-181a blocked the biosynthesis of TGF-β receptor 1 (TGFBR1) and TGF-β receptor associated protein 1 (TGFBRAP1), suggesting that miR-181a may enhance immune recognition by attenuating the immunosuppressive TGF-β pathway [95]. In neuroblastoma cells, miR-335 inhibited the non-canonical TGF-β pathway by targeting mitogen-activated protein kinase 1 (MAPK1) and Rho-associated coiled-coil-containing protein (ROCK1), leading to the suppression of cell migration and invasion [96]. miR-148a also reduced ROCK1 expression in gastric cancer and suppressed tumor cell invasion and metastasis [97]. Whether these TS-miRNAs increase immune recognition of cancer cells *in vivo* awaits further study. IL-10 expression is regulated by miR-106a in the T-cell leukemia Jurkat cell line [98]. Although it has not been tested *in vivo* if miR-106a enhances immune recognition, miR-106a suppressed the growth of glioma cells lines U87 and SHG44 and correlated inversely with glioma tumor grade [99]. Several additional lines of evidence support the notion that TS-miRNAs can target the STAT3 pathway to alleviate immune suppression: (i) let-7a overexpression in hepatoma cells suppressed cell proliferation through STAT3, (ii) miR-17-5p and miR-20a abrogated the immune-suppressive effects of MDSCs *in vivo* through the regulation of STAT3 and (iii) miR-93 inhibited tumor development and metastasis in mouse xenografts, at least in part by attenuating the TGF-β and/or STAT3 pathways [100–102]. Collectively, these findings indicate that altered miRNA expression is associated with failure of the immune system to recognize and eradicate cancer cells at an early time. Accordingly, increased expression of TS-miRNAs that enhance immune-suppressive mechanisms could strengthen the immune system to recognize cancer cells and eliminate them.

## 6. TS-miRNAs that Suppress Invasion and Metastasis

Metastasis is often associated with changes in cell morphology and adherence to other cells in the ECM. For instance, during epithelial-to-mesenchymal transition (EMT) or transformation, cancer cells can lose E-cadherin, a protein that strongly represses transformation [103]. This transition is accompanied by increased expression of N-, P- and T-cadherins in cancer cells, which can promote tumor cell invasion, even if E-cadherin function is unaltered [104]. Elevated levels of transcriptional repressors of E-cadherin, such as Snail, Twist and zinc finger E-box binding homeobox (ZEB1/2), are also responsible for EMT [105]. MMPs also facilitate or enhance tumor invasion and metastasis by degrading the extracellular matrix [75,106]. Concurrent inhibition of c-Met and VEGF signaling was recently shown to suppress tumor invasion and metastasis [107]. In addition, factors, such as HMGA1, Bcl-2, SirT1, N-Ras, K-RAS, Ezrin, Mucin 4, E2F and metastasis-associated gene (MTA1), are also involved in tumor invasion and metastasis [108–116]. These factors enhance invasion and metastasis, although they can also play roles in other steps of tumorigenesis.

Several TS-miRNAs have been identified as having an anti-metastatic and anti-invasion influence. miR-9 upregulated E-cadherin and downregulated Snail through its direct effect on NF-κB, leading to inhibition of melanoma growth and metastasis *in vivo* [117]. miR-200c inhibited EMT and cancer cell migration by suppressing ZEB1 and ZEB2, two suppressors of E-cadherin, as explained above [118]. In breast cancer and melanoma cells, miR-340 and miR-34a lowered c-Met (an inducer of MMP-2 and MMP-9) and, thus, suppressed tumor cell migration and invasion [81,119]. miR-29b inhibited MMP-2 expression, leading to an attenuation of the invasive capacity of HCC cells *in vitro* [73], while in glioma cell lines and tumors, miR-205 was found to inhibit invasion by targeting VEGF-A [78,82]. In breast cancer cells, miR-145 suppressed growth and invasion through VEGF and N-Ras, while overexpression of miR-183 resulted in reduced migration and invasion, an effect that was attributed in part to the downregulation of villin 2 (Ezrin) [46,120]. Repression of HMGA1 by miR-296 suppressed invasion of prostate cancer [41], while expression of K-Ras, a promoter of migration and invasion, was blocked by miR-96 in pancreatic cancer cells and by miR-216 in nasopharyngeal carcinoma [52,121]. In glioma cells, overexpression of miR-195, a repressor of E2F3 and Cyclin D3, induced cell cycle arrest and inhibited cell invasion, while in endometrial cancer cells, miR-30c repressed MTA1 production and blocked cell proliferation, migration and invasion [122,123]. In pancreatic cancer, miR-150 was identified as a potential tumor suppressor and correlated inversely with the levels of Mucin 4, a factor that promotes growth, invasion and metastasis of cancer cells [124]. miR-1 was found to target the oncogene purine nucleoside phosphorylase (PNP) in prostate cancer, thus suppressing cell migration and invasion, in agreement with the effects of PNP silencing in prostate cancer lines PC3 and DU145 [125].

Taken together, suppression of TS-miRNAs in cancer cells promotes the expression of many pro-invasion and pro-metastatic proteins. Overexpression of some of these miRNAs in cancer cells has shown that they are capable of suppressing tumor invasion and metastasis *in vivo*.

## 7. TS-miRNAs in Cancer Therapeutics

As highlighted above and listed in Table 1, several miRNAs were identified as tumor suppressors, because they inhibit one or more cancer traits. These miRNAs are often dysregulated in cancers, and several studies demonstrated that their restoration attenuates tumorigenesis both in cultured cells and *in vivo*. The discovery of TS-miRNAs has prompted researchers, clinicians and pharmaceutical companies to consider TS-miRNA-based approaches in cancer therapy. Although interventions to modulate a single miRNA or sets of miRNAs alone are unlikely to cure cancer, they are being actively considered in combination with other treatment regimens, including chemotherapy and radiotherapy. Just as miRNA antagonists are being developed to target oncomiRs elevated in cancer, the development of mimics for the downregulated TS-miRNAs is also underway [126,127]. Moreover, interventions to restore lost TS-miRNA activity may hold greater promise than efforts to antagonize endogenous miRNAs, since mimics resemble the native molecules and can control the same range of genes and pathways as those depleted in cancer cells. Mimics of the widely known TS-miRNA miR-34a were investigated for their therapeutic potential in multiple myeloma; the study concluded that miR-34a has therapeutic activity in preclinical models and prompted the development of miR-34a-based treatment in multiple myeloma patients [128]. Another study found that low expression of miR-148a was associated with poor survival rates in patients with advanced colorectal cancer, suggesting that miR-148a may be used to improve colorectal cancer therapy [129]. Pharmaceutical companies, such as Rosetta Genomics, Mirna Therapeutics, Alnylam and Santaris Pharma, have programs dedicated to the development and improvement of miRNA-based cancer diagnosis and therapy [130]. Future clinical and pharmaceutical studies will likely expand miRNA-based therapy to include many more miRNA candidates, not only for cancer treatments, but for other diseases as well.

## 8. Concluding Remarks and Perspectives

**Figure 1:**
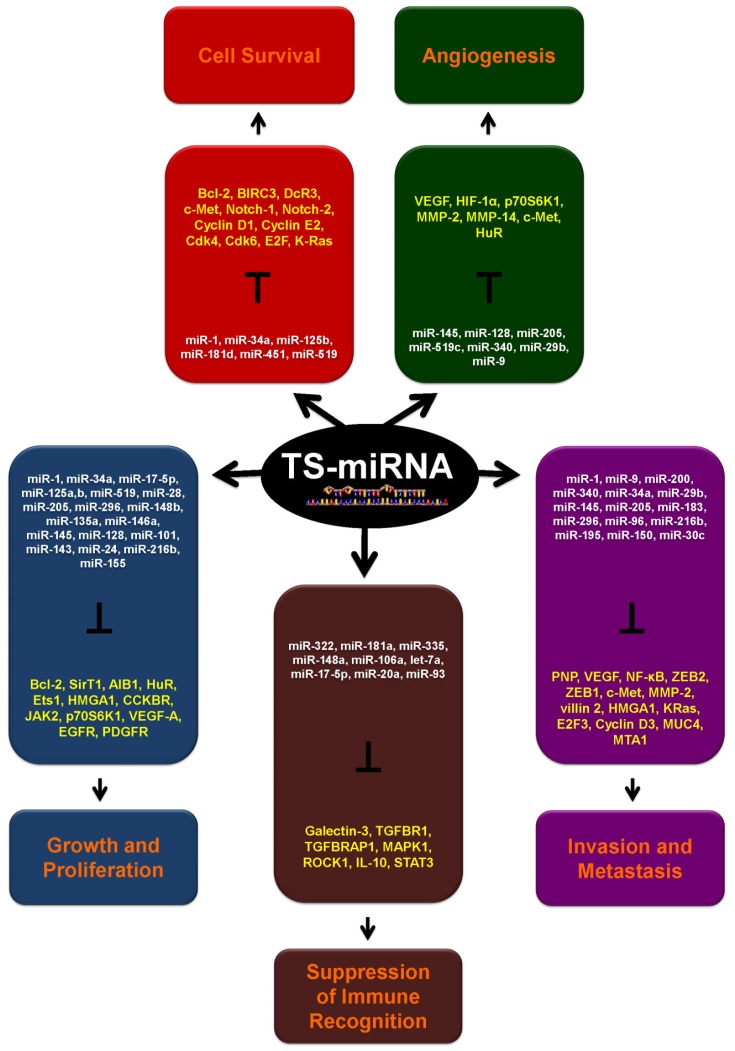
TS-miRNAs and their target mRNAs encoding proteins that enable cancer traits. Boxes depict five major cancer traits: Growth and Proliferation (blue), Cell Survival (red), Angiogenesis (green), Suppression of Immune Recognition (brown) and Invasion and Metastasis (purple). For each cancer trait, TS-miRNAs are indicated in white, the pro-oncogenic proteins encoded by mRNAs that are targets of TS-miRNAs are indicated in yellow and the cancer traits in orange.

As shown in Figure 1, TS-miRNAs are capable of regulating multiple cancer traits. A few miRNAs have entered preclinical and clinical studies, but many more *in vivo* studies are needed in order to determine which TS-miRNAs are effective for inclusion in specific cancer treatments. The miRNAs studied thus far only comprise a small fraction of the miRNAs discovered by high-throughput approaches, such as RNA sequencing [131–133]. Studies that consider TS-miRNAs together with radiotherapy or chemotherapeutic drugs may also be beneficial in cancer treatments. In this regard, altered miRNA expression in cancer cells can also influence the mechanisms of drug resistance, as low miRNA levels in cancer cells reduced the sensitivity to chemotherapeutic agents, while miRNA restoration or overexpression increased it (as shown for let-7a, miR-34a and miR-200c, miR-128 and miR-125b, reviewed in reference [134]). In addition, studying miRNA signatures (oncomiRs and TS-miRs) linked to drug responses can enhance drug efficacy and minimize toxicity. Circulating cancer-related miRNAs, including TS-miRNAs, are gaining preclinical and clinical attention, and many of them originate from circulating tumor cells [135–138]. Circulating miRNAs can correlate with tumor progression, as shown in prostate cancer patients [139], and can help with the design of therapeutic regimes. A number of clinical studies are already underway ( ClinicalTrials.gov) [140]. As our knowledge of TS-miRNAs continues to expand, we anticipate an escalation in interest in this class of microRNAs for cancer diagnosis and therapy.

## Figures and Tables

**Table 1:** Tumor-suppressive miRNAs (TS-miRNAs) involved in oncogenic traits. The table lists the TS-miRNAs discussed in this review, categorized by cancer trait (column 1), the oncogenic proteins encoded by TS-miRNA target mRNAs (column 2), the tumors in which TS-miRNAs have been characterized (column 3) and representatively referenced (column 4).

TS-miRNAs	Cancer-related proteins encoded by mRNAs that are TS-miRNA targets	Cancer models implicating TS-miRNAs	References
**Cell growth and proliferation**			
miR-34a	Bcl-2, SirT1	breast cancer, glioma stem cells (GSCs)	[20]
miR-17-5p	AIB1	breast cancer	[24]
miR-125a	HuR	breast carcinoma cell lines	[27,28]
miR-519	HuR	cervical, colon, ovarian, lung, kidney cancer	[29,30]
miR-125b	Ets1, Bcl-2	breast cancer, hepatocellular carcinoma	[31,32]
miR-28	Cyclin D1	colorectal cancer	[39]
miR-296	HMGA1	prostate cancer	[41]
miR-148b	CCKBR	gastric cancer	[42]
miR-135a	JAK2	gastric cancer	[43]
miR-146a	FADD, EGFR, ROCK1, NOTCH1, CXCR4, TRAF6	glioblastoma and breast, pancreatic, gastric, prostate cancer glioblastoma	[44,45]
miR-145	VEGF-A, N-Ras, 70S6K1, FSCN1	Kaposi’s sarcoma, T lymphocyte Jurkat cells, leukemia, extranodal NK/T cell lymphoma	[46,47]
miR-128	EGFR, PDGFRα	colon and breast cancer, esophageal squamous cell carcinoma	[48]
miR-101	EZH2	glioma	[49]
miR-143, miR-145	Bcl-2, Top2A, PRC1, Plk1	bladder transitional cell carcinoma	[50]
miR-24	S100A8	liposarcoma	[51]
miR-216b	K-Ras	laryngeal squamous cell carcinoma	[52]
**Cell survival**			
miR-34a	Bcl2, SirT1, BIRC3, DcR3, c-Met, Notch-1, Notch-2, Cyclin D1, Cyclin E2, Cdk4, Cdk6, E2F	brain tumors, glioma stem cell lines, breast, colon, pancreatic cancer	[20,61,62, 65–67]
miR-181d	K-Ras, Bcl-2	glioma	[68]
miR-451	Cyclin D1, Bcl-2, Akt1, MMP-2, MMP-9	glioblastoma	[69]
**Angiogenesis**			
miR-145	VEGF-A, N-Ras, p70S6K1	colon and breast cancer	[46,47]
miR-128	p70S6K1	glioma	[77]
miR-205	VEGF-A	glioma	[78]
miR-519c	HIF-1α, HuR	lung, breast, cervical, colon, ovarian cancer	[27,29,30, 70,71,80]
miR-340	c-Met	breast cancer	[81]
miR-29b	MMP-2	hepatocellular carcinoma	[82]
miR-9	MMP-14	neuroblastoma	[83]
**Suppressors of immune recognition**			
miR-322	galectin-3	breast, lung, prostate, kidney cancer	[94]
miR-181a	TGFBR1, TGFBRAP1	mesenchymal stem cells	[95]
miR-335	MAPK1, ROCK1	neuroblastoma	[96]
miR-148a	ROCK1	gastric cancer	[97]
miR-106a	IL-10, E2F1	T lymphocyte Jurkat cells, glioma	[98,99]
let-7a	STAT3	hepatocellular carcinoma	[100]
miR-17-5p	STAT3	MDSCs	[101]
miR-20a	STAT3	MDSCs	[101]
miR-93	Genes of the TGF-β and/or STAT3 pathway	breast cancer	[102]
**Invasion and metastasis**			
miR-9	PNP	Melanoma	[117,125]
miR-1	NF-κB	prostate cancer	
miR-200	ZEB1	colorectal cancer	[118]
miR-340	c-Met	breast cancer	[81]
miR-34a	c-Met	breast cancer, melanoma	[119]
miR-29b	MMP-2	hepatocellular carcinoma	[82]
miR-145	VEGF, N-Ras	breast cancer	[46]
miR-205	VEGF-A	glioblastoma	[78]
miR-183	villin 2 (Ezrin),	breast cancer	[120]
miR-296	HMGA1	prostate cancer	[41]
miR-96	KRAS	pancreatic cancer	[121]
miR-216b	KRAS	nasopharyngeal carcinoma	[52]
miR-195	E2F3, CCND3	glioblastoma	[122]
miR-150	MUC4	pancreatic cancer	[124]
miR-30c	MTA1	endometrial cancer	[123]
